# Data provenance tracking and reporting in a high-security digital research environment.

**DOI:** 10.23889/ijpds.v7i3.1909

**Published:** 2022-08-25

**Authors:** Bernhard Scheliga, Milan Markovic, Helen Rowlands, Artur Wozniak, Katie Wilde, Jessica Butler

**Affiliations:** 1 University of Aberdeen

## Objectives

We designed the Safe Haven Provenance (SHP) ontology for representing provenance information about data, users, and activities within high-security environments as knowledge graphs. The work was based on a case study of the Grampian Data Safe Haven (DASH) which holds and processes medical records for 600,000 people in Scotland. The SHP ontology was designed as an extension to the standard W3C PROV-O ontology. The auditing capabilities of our approach were evaluated against a set of transparency requirements through a prototype interactive dashboard.

## Approach

We demonstrated the ability of the SHP ontology to document the workflow within DASH: capturing the extraction and anonymisation process using a structured vocabulary of entities (e.g. datasets), activities (e.g. linkage, anonymisation) and agents (e.g. analysts, data owners). Two provenance reporting templates were designed following interviews with DASH staff and clinical researchers: 1) a detailed report for use within DASH for quality assurance, and 2) a summary report for researchers that was safe for public release. Using a prototype data-linkage project, we formalised queries for report generation, and demonstrated use of automated rules for error detection (e.g., data discrepancies) using the structure of the SHP knowledge graphs. All of the project outputs are available under an open-source license..

## Results

We demonstrated the ability of the SHP ontology to document the workflow within DASH: capturing the extraction and anonymisation process using a structured vocabulary of entities (e.g. datasets), activities (e.g. linkage, anonymisation) and agents (e.g. analysts, data owners). Two provenance reporting templates were designed following interviews with DASH staff and clinical researchers: 1) a detailed report for use within DASH for quality assurance, and 2) a summary report for researchers that was safe for public release. Using a prototype data-linkage project, we formalised queries for report generation, and demonstrated use of automated rules for error detection (e.g., data discrepancies) using the structure of the SHP knowledge graphs. All of the project outputs are available under an open-source license.

## Conclusion

This project lays a foundation for more transparent high-quality research using public data for health care and innovation. The SHP ontology is extendible for different domains and potentially represents a key component for further automation of provenance capture and reporting in high-security research environments.

